# Supplementary material for: Extracellular vesicles from human plasma and serum are carriers of extravesicular cargo—Implications for biomarker discovery
Source: PLoS One. 2020 Aug 19;15(8):e0236439. doi: 10.1371/journal.pone.0236439 (PMC7446890; doi:10.1371/journal.pone.0236439)
Supplement: S1 Raw images — (PDF) [file pone.0236439.s001.pdf]

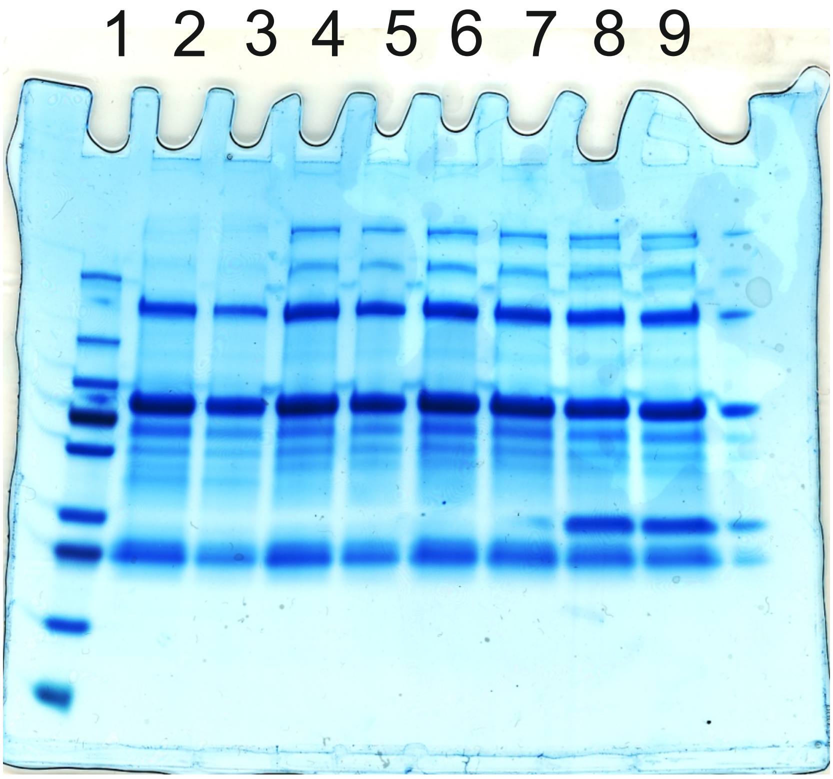

SDS-PAGE
Samples: 1) Protein standard, Precision Plus Protein Kaleidoscope (Bio-Rad), 2) Serum EV1, 3) Serum EV2, 4) ACD EV1, 5) ACD EV2, 6) Citrate EV1, 7) Citrate EV2, 8) EDTA EV1, 9) EDTA Ev2
Total of 5 x 10E10 particles of samples were run on 4-20% gradient gel (Bio-Rad) in reducing condition.
The gel was stained with Coomassie Brilliant blue, and scanned.

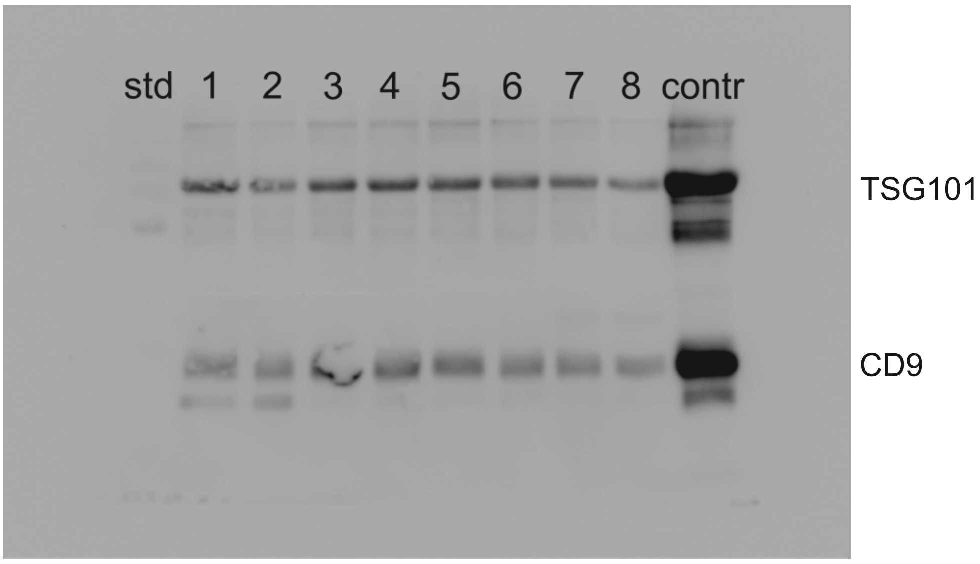

Western blotting
Samples: STD=Protein standard, Precision Plus Protein Kaleidoscope (Bio-Rad), 1) Serum EV1, 2) Serum EV2, 3) ACD EV1, 4) ACD EV2, 5) Citrate EV1, 6) Citrate EV2, 7) EDTA EV1, 8) EDTA EV2, contr=lyophilized exosomes from PC-3 cell line (Hansa BioMed)
Total of 5 x 10E10 particles of samples were run on 4-20% gradient gel (Bio-Rad) in reducing condition.
Proteins were transfered to PVDF membrane and incubated with anti-TSG101 and anti-CD9 antibodies.
