# Supplementary figures and images for: Extracellular vesicles from human plasma and serum are carriers of extravesicular cargo—Implications for biomarker discovery
Source: PLoS One. 2020 Aug 19;15(8):e0236439. doi: 10.1371/journal.pone.0236439 (PMC7446890; doi:10.1371/journal.pone.0236439)

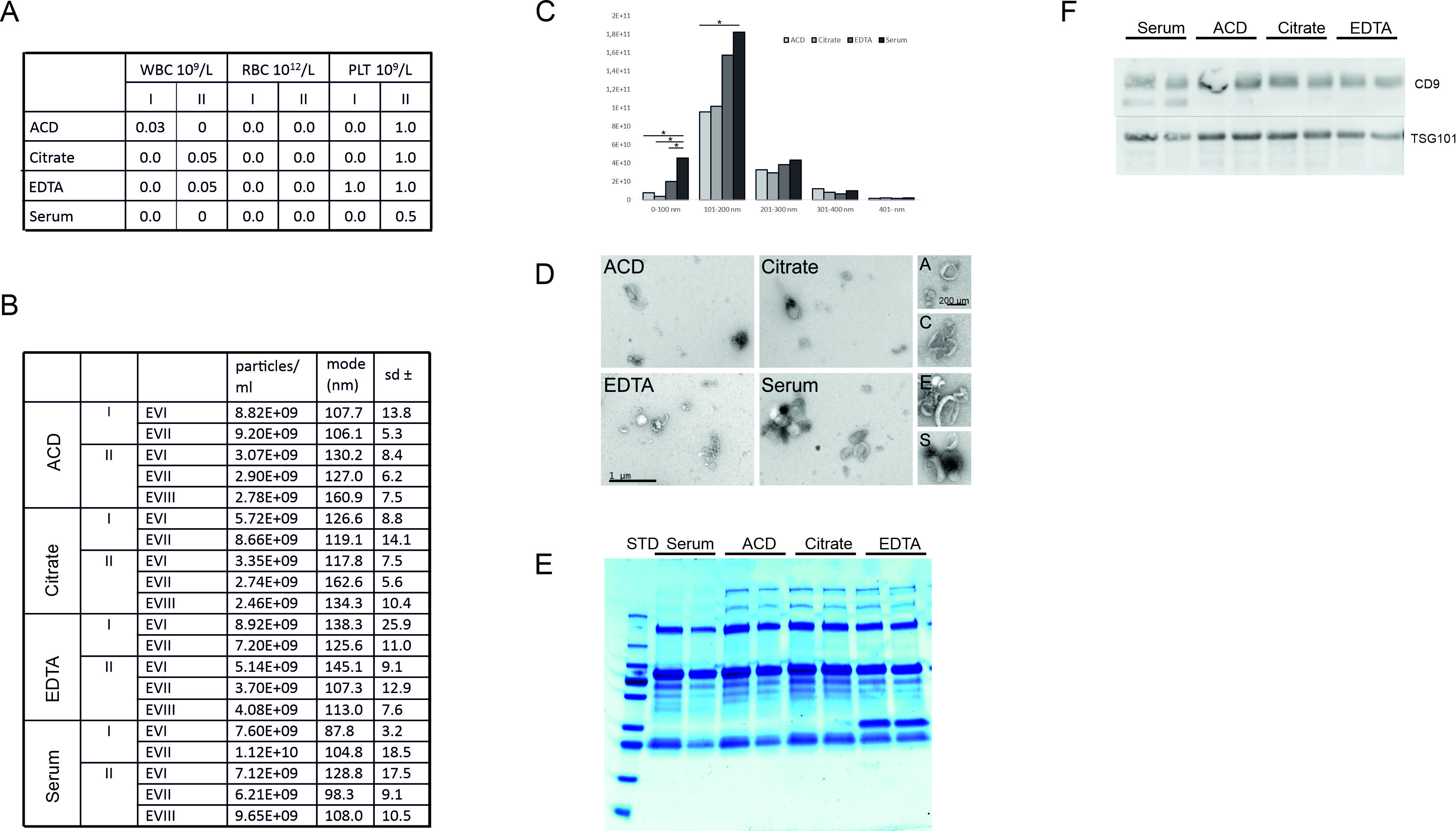

Supplement: S1 Fig — (JPG) [file pone.0236439.s002.jpg]
