# Supplementary material for: Extracellular vesicles from human plasma and serum are carriers of extravesicular cargo—Implications for biomarker discovery
Source: PLoS One. 2020 Aug 19;15(8):e0236439. doi: 10.1371/journal.pone.0236439 (PMC7446890; doi:10.1371/journal.pone.0236439)
Supplement: S1 Table — (DOCX) [file pone.0236439.s003.docx]

S1 table. Workflow for the analyses.

|  | | | Set I Helsinki | | | Set II Helsinki | Set III Budabest | |
| --- | --- | --- | --- | --- | --- | --- | --- | --- |
| Donors | N= | | 10 (pooled) | | | 10 (pooled) | 3 | |
|  | Age (mean) | | 45 y | | | 32 y | 30y | |
|  | Female % | | 80% | | | 70% | 67% | |
|  | Volume (ml) per replicate | plasma | | 10 ml | 10.5 ml | | | 100 µl |
|  |  | serum | | 10 ml | 7.5 ml | | | 100 µl |
| Isolation | | | Ultracentifugation:  Dilution 1:1 PBS  110 000 g 90 min +4˚C  Washing step with 20 ml PBS 110 000 g 90 min +4˚C | | | Ultracentifugation:  Dilution 1:1 PBS  110 000 g 90 min +4˚C  Washing step with 20 ml PBS 110 000 g 90 min +4˚C | SEC | |
| Technical replicates | | | 2 | | | 3 | 3 | |
| Methods | | | Remnant analysis  NTA  Apogee (CD61, CD235a, AnnexinV  SDS-PAGE  Western blotting | | | Remnant analysis  NTA  Apogee (CD61, CD235a, AnnexinV  Proteomics | Flu-SEC (CD61) | |
