# Supplementary material for: Extracellular vesicles from human plasma and serum are carriers of extravesicular cargo—Implications for biomarker discovery
Source: PLoS One. 2020 Aug 19;15(8):e0236439. doi: 10.1371/journal.pone.0236439 (PMC7446890; doi:10.1371/journal.pone.0236439)
Supplement: S2 Table — (DOCX) [file pone.0236439.s004.docx]

S2 table. Apogee (Kruskall-Wallis test followed by Dunns test)

|  | Mean events/µl (±SD) | | | | Overall test  (*p*-value) | Pairwise test (*p*-value) | | | | | |
| --- | --- | --- | --- | --- | --- | --- | --- | --- | --- | --- | --- |
|  | **ACD** | **Citrate** | **EDTA** | **Serum** |  | ACD vs Citrate | ACD vs EDTA | ACD vs Serum | EDTA vs Citrate | EDTA vs Serum | Citrate vs Serum |
| CD61 | 3296  (±426) | 6332  (±971) | 4022 (±4022) | 11546 (±4027) | 0.023* | 0.42 | 0.428 | 0.005 | 0.213 | 0.042 | 0.428 |
| Annexin V | 5326 (±1120) | 7718  (±837) | 6582 (±1293) | 16090 (±4505) | 0.033* | 0.113 | 0.428 | 0.005 | 0.428 | 0.042 | 0.213 |
| CD235a | 5205  (±409) | 6098 (±1629) | 6743  (±590) | 6951 (±2253) | 0.459 | N/A | N/A | N/A | N/A | N/A | N/A |
