# Supplementary material for: Extracellular vesicles from human plasma and serum are carriers of extravesicular cargo—Implications for biomarker discovery
Source: PLoS One. 2020 Aug 19;15(8):e0236439. doi: 10.1371/journal.pone.0236439 (PMC7446890; doi:10.1371/journal.pone.0236439)
Supplement: S3 Table — (DOCX) [file pone.0236439.s005.docx]

S3 table. Flu-SEC (Tukey HDD)

| Tukey´s multiple comparisons test | Mean diff. | 95.00% CI of diff. | Significant? | Adjusted *p*-value |
| --- | --- | --- | --- | --- |
| EDTA vs. citrate | -766671 | -1519290 to -14052 | Yes | 0.046 |
| EDTA vs. ACD | -145508 | -898127 to 607110 | No | 0.923 |
| EDTA vs. serum | -5810568 | -6563186 to -5057949 | Yes | <0.001 |
| Citrate vs. ACD | 621163 | -131456 to 1373781 | No | 0.110 |
| Citrate vs. serum | -504397 | -5796515 to -4291278 | Yes | <0.001 |
| ACD vs. serum | -5665059 | -6417678 to -4912441 | Yes | <0.001 |
|  |  |  |  |  |
| Test details | **Mean 1** | **Mean 2** | **Fold change** |  |
| EDTA vs. citrate | 142668 | 909339 | -6.4 |  |
| EDTA vs. ACD | 142668 | 288177 | -2.0 |  |
| EDTA vs. serum | 142668 | 5953236 | -41.7 |  |
| Citrate vs. ACD | 909339 | 288177 | 3.2 |  |
| Citrate vs. serum | 909339 | 5953236 | -6.5 |  |
| ACD vs. serum | 288177 | 5953236 | -20.7 |  |
