# Supplementary material for: Extracellular vesicles from human plasma and serum are carriers of extravesicular cargo—Implications for biomarker discovery
Source: PLoS One. 2020 Aug 19;15(8):e0236439. doi: 10.1371/journal.pone.0236439 (PMC7446890; doi:10.1371/journal.pone.0236439)
Supplement: S6 Table — (DOCX) [file pone.0236439.s008.docx]

S6 table. Gene ontology analysis of albumin-depleted proteins with *p*-value ≤0.05 (Holm-Bonferroni corrected)

| Cellular component Bonferroni method *p*≤0.05 | | | |
| --- | --- | --- | --- |
| Exosomes | YWHAG; ACTB; SERPINA1; AHSG; A2M; APOA1; APOA2; APOD; APOE; APOL1; C4BPA; C4BPB; CALM1; CALM2; CALM3; CD5L; CP; CLU; CFL1; C1QB; C1QC; C1R; C3; C4A; C4B; CFH; DCD; ECM1; FGA; FGB; FGG; FCN2; FCN3; LGALS3BP; GSN; GAPDH; HP; HPR; HBA1; HBA2; HBB; HBD; HPX; JCHAIN; ITGA2B; ITIH4; JUP; KRT10; KRT12; KRT13; KRT14; KRT16; KRT17; KRT18; KRT19; KRT20; KRT9; KRT77; KRT2; KRT3; KRT4; KRT5; KRT6A; KRT6B; KRT7; KRT75; KRT8; MASP1; MYL6; MYL12B; POTEF; S100A9; TF; SH3BGRL3; TTR; TPM3; TPM4; PROS1; VTN; AZGP1; | | |
| Cytoplasm | YWHAG; ACTB; SERPINA1; AHSG; APOA1; APOA2; APOE; APOH; CALM1; CALM2; CALM3; CASP14; CIT; CLU; CFL1; C1QA; C1QC; C3; DCD; KHSRP; FGA; FGB; FGG; GSN; GFAP; GAPDH; HP; HBA1; HBA2; HBB; HBD; ITGA2B; JUP; KRT36; KRT10; KRT12; KRT13; KRT14; KRT16; | | |
| Extracellular | ACTB; ORM1; ORM2; SERPINA1; AHSG; A2M; APOA1; APOA2; APOC3; APOD; APOE; APOL1; APOH; C4BPA; C4BPB; CASP14; CD5L; CP; CLU; CFL1; C1QA; C1QB; C1QC; C1R; C1S; C3; C4A; CFH; DCD; ECM1; FGA; FGB; FGG; FCN2; FCN3; LGALS3BP; GSN; GAPDH; HP; HPR; HBA1; HBB; HBD; HPX; JCHAIN; ITIH4; JUP; KRT17; KRT18; KRT9; KRT86; KRT2; KRT8; MASP1; OIT3; PPBP; CFP; S100A9; F2; TF; TTR; TPM3; TPM4; WDR48; GC; PROS1; VTN; AZGP1; | | |
| Extracellular region | ORM1; SERPINA1; AHSG; A2M; APOA1; APOA2; APOC3; APOD; APOE; C4BPA; C4BPB; CALM1; CALM2; CALM3; CD5L; CP; C1QA; C1QB; C1QC; C1R; C1S; C3; C4A; DCD; FGA; FGB; FGG; FCN3; GSN; HP; HPX; JCHAIN; ITIH4; MASP1; PPBP; CFP; F2; TF; TTR; GC; PROS1; VTN; AZGP1; | | |
| Lysosome | YWHAG; ACTB; ORM1; SERPINA1; AHSG; A2M; APOA1; APOA2; APOD; APOH; C4BPA; CALM1; CALM2; CALM3; CASP14; CP; CLU; CFL1; C4A; C4B; CFH; DCD; FGA; LGALS3BP; GSN; GAPDH; HP; HBA1; HBA2; HBB; HBD; HPX; ITGA2B; ITIH4; JUP; S100A9; TF; TTR; TPM3; TPM4; WDR48; VTN; AZGP1; | | |
| Cytoskeleton | YWHAG; ACTB; APOA1; APOE; CLU; CFL1; DCD; FGA; FGB; FGG; GSN; GAPDH; ITGA2B; JUP; KRT10; KRT12; KRT13; KRT14; KRT16; KRT18; KRT9; KRT77; KRT2; KRT5; KRT8; MYL6; MYL12A; PPBP; TTR; TPM3; TPM4; | | |
| Extracellular space | ORM1; ORM2; SERPINA1; AHSG; APOA1; APOC3; APOD; APOE; APOL1; APOH; CD5L; CP; CLU; CFH; FGA; FGB; FGG; LGALS3BP; HPX; MASP1; CFP; F2; VTN; | | |
| Intermediate filament | GFAP; KRT10; KRT14; KRT16; KRT17; KRT18; KRT19; KRT20; KRT86; KRT2; KRT3; KRT4; KRT5; KRT6A; KRT7; KRT8; | | |
| Very-low-density lipoprotein particle | APOA1; APOA2; APOC3; APOE; APOL1; APOH; | | |
| Platelet alpha granule lumen | SERPINA1; A2M; FGA; FGB; FGG; PPBP; | | |
| Spherical high-density lipoprotein particle | APOA1; APOA2; APOC3; CLU; HPR; | | |
| High-density lipoprotein particle | APOA1; APOA2; APOE; APOL1; APOH; | | |
| Keratin filament | KRT14; KRT18; KRT3; KRT5; | | |
| Chylomicron | APOA2; APOC3; APOE; APOH; | | |
| Hemoglobin complex | HBA1; HBA2; HBB; HBD; | | |
| Fibrinogen complex | FGA; FGB; FGG; | | |
| Platelet alpha granule | FGA; FGB; FGG; | | |
| Complement component C1 complex | C1QA; C1QB; | | |
| Molecular function Bonferroni method *p*≤0.05 | | |  |
| Structural constituent of cytoskeleton | | ACTB; GSN; KRT14; KRT16; KRT17; KRT18; KRT19; KRT85; KRT86; KRT3; KRT4; KRT7; MYL6; MYL12B; TPM4; |  |
| Transporter activity | | APOA1; APOA2; APOC3; APOD; APOE; APOH; HP; HBA1; HBA2; HBB; HBD; HPX; TF; TTR; GC; |  |
| Structural molecule activity | | GFAP; KRT36; KRT10; KRT12; KRT13; KRT9; KRT77; KRT5; KRT6A; KRT6B; KRT75; KRT8; |  |
| Complement activity | | C4BPA; C4BPB; CLU; C1QA; C1QB; C1QC; C1R; C1S; C3; C4A; CFP; |  |

| Biological process Bonferroni method *p*≤0.05 | |
| --- | --- |
| Cell growth and/or maintenance | ACTB; CFL1; ECM1; GSN; GFAP; KRT36; KRT10; KRT12; KRT13; KRT14; KRT16; KRT17; KRT18; KRT19; KRT9; KRT85; KRT86; KRT77; KRT2; KRT3; KRT4; KRT5; KRT6A; KRT6B; KRT7; KRT75; KRT8; MYL6; MYL12B; TMSB4X; TPM3; TPM4; VTN; |
| Immune response | ORM1; ORM2; C4BPA; C4BPB; CD5L; CLU; C1QA; C1QB; C1QC; C1R; C1S; C3; C4A; CFH; DCD; FCN2; FCN3; LGALS3BP; HP; HPR; JCHAIN; CFP; AZGP1; |
