# Supplementary material for: Extracellular vesicles from human plasma and serum are carriers of extravesicular cargo—Implications for biomarker discovery
Source: PLoS One. 2020 Aug 19;15(8):e0236439. doi: 10.1371/journal.pone.0236439 (PMC7446890; doi:10.1371/journal.pone.0236439)
Supplement: S7 Table — (DOCX) [file pone.0236439.s009.docx]

S7 table. Gene ontology analysis of proteins shared with this study and NP protein corona studies with *p*-value ≤0.05 (Holm-Bonferroni corrected)

| Cellular component Bonferroni method *p*≤0.05 | |
| --- | --- |
| Exosomes | A2M; APOA2; APOD; APOE; APOL1; AZGP1; C1QB; C1QC; C3; C4A; C4BPA; CFH; CLU; CP; FGA; FGB; FGG; HP; ITIH4; SERPINA1; TF; VTN; ACTB; AHSG; APOA1; C1R; C4B; CD5L; FCN3; GAPDH; HBA1; HBB; HBD; HPR; HPX; ITGA2B; JCHAIN; KRT1; KRT10; KRT12; KRT13; KRT14; KRT16; KRT17; KRT18; KRT19; KRT2; KRT3; KRT4; KRT5; KRT6A; KRT6B; KRT75; KRT77; KRT8; KRT9; LGALS3BP; MYL6; TTR; DCD; MASP1; PROS1 |
| Extracellular region | A2M; APOA2; APOC3; APOD; APOE; AZGP1; C1QB; C1QC; C1S; C3; C4A; C4BPA; CFP; CP; FGA; FGB; FGG; GC; HP; ITIH4; SERPINA1; TF; VTN; AHSG; APOA1; C1QA; C1R; CD5L; F2; FCN3; HPX; JCHAIN; ORM1; TTR; DCD; MASP1; PROS1 |
| Extracellular | A2M; APOA2; APOC3; APOD; APOE; APOH; APOL1; AZGP1; C1QB; C1QC; C1S; C3; C4A; C4BPA; CFH; CFP; CLU; CP; FGA; FGB; FGG; GC; HP; ITIH4; SERPINA1; TF; VTN; ACTB; AHSG; APOA1; C1QA; C1R; CD5L; F2; FCN3; GAPDH; HBA1; HBB; HBD; HPR; HPX; JCHAIN; KRT17; KRT18; KRT2; KRT8; KRT9; LGALS3BP; ORM1; ORM2; TTR; DCD; MASP1; PROS1 |
| Intermediate filament | KRT1; KRT10; KRT14; KRT16; KRT17; KRT18; KRT19; KRT2; KRT3; KRT4; KRT5; KRT6A; KRT8 |
| Cytoskeleton | APOE; CLU; FGA; FGB; FGG; ACTB; APOA1; GAPDH; ITGA2B; KRT1; KRT10; KRT12; KRT13; KRT14; KRT16; KRT18; KRT2; KRT5; KRT77; KRT8; KRT9; MYL6; TTR; DCD |
| Extracellular space | APOC3; APOD; APOE; APOH; APOL1; CFH; CFP; CLU; CP; FGA; FGB; FGG; SERPINA1; VTN; AHSG; APOA1; CD5L; F2; HPX; LGALS3BP; ORM1; ORM2; MASP1 |
| Lysosome | A2M; APOA2; APOD; APOH; AZGP1; C4A; C4BPA; CFH; CLU; CP; FGA; HP; ITIH4; SERPINA1; TF; VTN; ACTB; AHSG; APOA1; C4B; GAPDH; HBA1; HBB; HBD; HPX; ITGA2B; LGALS3BP; ORM1; TTR; DCD |
| Very-low-density lipoprotein particle | APOA2; APOC3; APOE; APOH; APOL1; APOA1 |
| Spherical high-density lipoprotein particle | APOA2; APOC3; CLU; APOA1; HPR |
| High-density lipoprotein particle | APOA2; APOE; APOH; APOL1; APOA1 |
| Keratine filament | KRT14; KRT18; KRT3; KRT5 |
| Chylomicron | APOA2; APOC3; APOE; APOH |
| Platelet alpha granule lumen | A2M; FGA; FGB; FGG; SERPINA1 |
| Fibrinogen complex | FGA; FGB; FGG |
| Hemoglobin complex | HBA1; HBB; HBD |
| Complement component C1 complex | C1QB; C1QA |
| Cytoplasm | APOA2; APOE; APOH; C1QC; C3; CLU; FGA; FGB; FGG; GC; HP; SERPINA1; TF; ACTB; AHSG; APOA1; C1QA; GAPDH; HBA1; HBB; HBD; ITGA2B; KRT1; KRT10; KRT12; KRT13; KRT14; KRT16; KRT17; KRT18; KRT19; KRT2; KRT3; KRT36; KRT4; KRT5; KRT6A; KRT6B; KRT75; KRT8; KRT9; MYL6; TTR; DCD; PROS1 |
| Intermediate-density lipoprotein particle | APOC3; APOE |
| Extracellular matrix | APOH; CFP; CLU; VTN; LGALS3BP |
| External side of plasma membrane | FGA; FGB; FGG; |

| Molecular function Bonferroni method *p*≤0.05 | |
| --- | --- |
| Transporter activity | APOA2; APOC3; APOD; APOE; APOH; GC; HP; TF; APOA1; HBA1; HBB; HBD; HPX; TTR |
| Structural molecule activity | KRT1; KRT10; KRT12; KRT13; KRT36; KRT5; KRT6A; KRT6B; KRT75; KRT77; KRT8; KRT9 |
| Complement activity | C1QB; C1QC; C1S; C3; C4A; C4BPA; CFP; CLU; C1QA; C1R |
| Structural constituent of cytoskeleton | ACTB; KRT14; KRT16; KRT17; KRT18; KRT19; KRT3; KRT4; MYL6 |
| Defense/immunity protein activity | AHSG; CD5L; ORM1; ORM2 |

| Biological process Bonferroni method *p*≤0.05 | |
| --- | --- |
| Cell growth and/or maintenance | VTN; ACTB; KRT1; KRT10; KRT12; KRT13; KRT14; KRT16; KRT17; KRT18; KRT19; KRT2; KRT3; KRT36; KRT4; KRT5; KRT6A; KRT6B; KRT75; KRT77; KRT8; KRT9; MYL6 |
| Immune response | AZGP1; C1QB; C1QC; C1S; C3; C4A; C4BPA; CFH; CFP; CLU; HP; C1QA; C1R; CD5L; FCN3; HPR; JCHAIN; LGALS3BP; ORM1; ORM2; DCD |
| Transport | APOA2; APOC3; APOD; APOE; APOH; APOL1; GC; TF; APOA1; HBA1; HBB; HBD; HPX; TTR; |
